# Synthesis of a hollow-structured flower-like Fe_3_O_4_@MoS_2_ composite and its microwave-absorption properties

**DOI:** 10.1039/d1ra02095a

**Published:** 2021-06-07

**Authors:** Guanghong Xiang, Mingyang Chen, Zhewei Ni, Yong Shen, Lihui Xu

**Affiliations:** School of Textile and Clothing, Shanghai University of Engineering Science Shanghai 201620 PR China shenyong@sues.edu.cn xulh0915@163.com +86-21-67791242

## Abstract

In order to realize the characteristics of new types of wave-absorbing materials, such as strong absorption, broad bandwidth, low weight and small thickness, a hollow-structured flower-like Fe_3_O_4_@MoS_2_ composite was successfully prepared by simple solvothermal and hydrothermal methods in this paper. The structural properties were characterized by X-ray diffraction, X-ray photoelectron spectroscopy, scanning electron microscopy (SEM) and transmission electron microscopy (TEM). Besides, the microwave properties and magnetic properties were measured using a vector network analyzer and *via* a hysteresis loop. SEM and TEM images revealed that MoS_2_ nanosheets grew on the surface of hollow nanospheres. The results showed that the composite exhibited excellent absorbing property. When the molar ratio of Fe_3_O_4_ and MoS_2_ was 1 : 18, the minimum reflection loss value reached −49.6 dB at 13.2 GHz with a thickness of 2.0 mm and the effective absorption bandwidth was 4.24 GHz (11.68–15.92 GHz). Meanwhile, the effective absorption in the entire X-band (8–12 GHz) and part of the C-band (4–8 GHz) and Ku-band (12–18 GHz) could be achieved by designing the sample thickness. In addition, the hollow structure effectively reduced the density of the material, which was in line with the current development trend of absorption materials. It could be predicted that the hollow core–shell structure composite has a potential application prospect in the field of microwave absorption.

## Abstract 2

A hollow-structured flower-like Fe_3_O_4_@MoS_2_ composite was synthesized. The minimum reflection loss value reached −49.6 dB at 13.2 GHz with a thickness of 2.0 mm and the effective absorbing bandwidth was 4.24 GHz (11.68–15.92 GHz).

## Graphical abstract

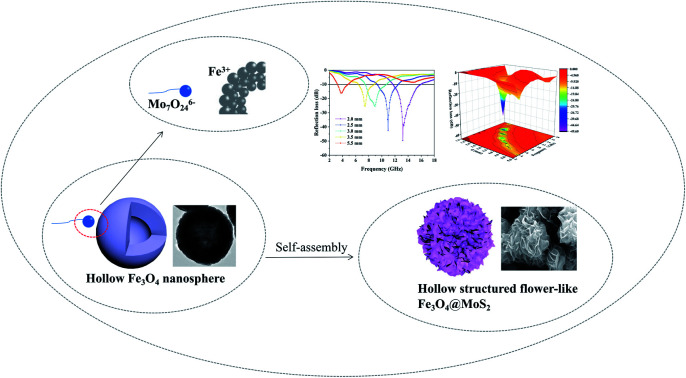

## 1. Introduction

With the advent of 5G, the relationship between wireless communication installations and human livelihoods is increasingly inseparable. Microwave radiation is becoming one of the most serious factors threatening human wellbeing,^1,2^ so it is urgent to settle the issue of electromagnetic contamination. Consequently, the exploration of electromagnetic absorbers is imbued with distinct real-world significance owing to the characteristics of strong absorption,^3^ low density,^4^ broad bandwidth and small thickness.^5^ Compared to the conventional electromagnetic shielding materials, use of microwave absorbers is regarded as a more efficacious method to dominate electromagnetic contamination.^6^ A good electromagnetic absorber must have impedance matching and attenuation characteristics to eliminate the problem of secondary reflection contamination and allow the electromagnetic energy to be converted into heat energy in the material.^7^ According to the fundamental formula, when the dielectric constant *ε* (*ε* = *ε*′ − *jε*′′) is equal to the permeability *μ* (*μ* = *μ*′ − *jμ*′′), the incident wave can totally enter the material without reflection.^8^ And the attenuation characteristics depend on the loss mechanism itself, which consists of magnetic loss, dielectric loss and conductivity loss. In addition, it has been reported that the morphology and construction have non-negligible effects on the absorbing properties.^9^

Traditional ferrite materials possess exclusive features of strong saturation magnetization, high complex permeability as well as being of low cost, which have been extensively applied in the field of microwave absorption.^10,11^ Ferrosoferric oxide (Fe_3_O_4_) as the simplest ferromagnetic substance has been widely used in dye degradation,^12^ biomedical applications,^13^ hydrogen storage,^14^ energy storage devices,^15^ electromagnetic absorbers,^16^*etc.* Especially in the domain of microwave absorption, natural resonance and eddy-current effect reveal unexceptionable magnetic loss in the high-frequency range. Nevertheless, the high permeability and low permittivity of the material mean it is difficult for it to satisfy the requirements of new wave-absorbing materials. In recent years, some combinations of ferromagnetic and graphene materials have been shown to exhibit excellent microwave absorption performance. The advantages are as follows. First, satisfactory impedance matching can be achieved by adjusting the electromagnetic parameters. Second, the completely different loss mechanisms produce beneficial synergistic effect. For example, Wang *et al.*^17^ investigated novel flower-like CoFe_2_O_4_@graphene complexes, in which hundreds of CoFe_2_O_4_ microspheres are combined using graphene as a medium to form a whole flower-like structure. The characteristic appearance utilized multi-polarization, hierarchical and synergistic effect to reveal an excellent miraculous absorbing capacity. The minimum reflection loss (RL) value reached −42 dB at 12.9 GHz with a thickness of 2.0 mm and the effective absorption (less than −10 dB) reached 4.59 GHz (11.2–15.79 GHz). Bateer *et al.*^18^ prepared NiFe_2_O_4_@RGO composite; the minimum RL value reached −27.7 dB at 9.2 GHz with a thickness of 3.0 mm and the effective absorbing bandwidth was 3.1 GHz. This material disperses well in nonpolar solvents, which has broad application prospects. However, the costly graphene and the high density of ferrite present new challenges. Therefore, it is necessary to find an alternative to graphene and to reduce the density of ferrite. This is because light and economical absorbing material has practical significance in industrial production.

The two-dimensional (2D) material molybdenum disulfide (MoS_2_) with graphene-like layered structure plays a significant role as a semiconductor,^19^ solid lubricant,^20^ catalyst,^21^*etc.* In the past few years, scientific researchers have discovered that MoS_2_ has superb dielectric loss property and is of low weight, which have made it a popular material in the preparation of lightweight electromagnetic absorbers. High-purity MoS_2_ can be prepared by means of hydrothermal treatment. Generally speaking, its morphology can be described as like a blooming flower which is made of flaky structures stacked on top of each other. This peculiar appearance will contribute to the amplification of specific surface area and consolidation of microwave absorbing capacity.^22^ Furthermore, the raw material is cheap and available and the yield is high, making it promising for replacement of graphene.

In this paper, hollow pellets of Fe_3_O_4_ were prepared by a facile solvothermal method in order to reduce the density. Then, MoS_2_ nanosheets were gradually grown on the hollow Fe_3_O_4_ microspheres by hydrothermal treatment and the obtained Fe_3_O_4_@MoS_2_ composite showed a hollow flower-like structure. The morphology and structure of Fe_3_O_4_ MPs, MoS_2_ MPs and Fe_3_O_4_@MoS_2_ MPs were investigated and their electromagnetic parameters and microwave absorption properties were explored. The results indicated that the composite possessed properties of good microwave absorption, wide bandwidth and low mass.

## 2. Experimental

### 2.1. Materials and reagents

Ferric chloride hexahydrate (FeCl_3_·6H_2_O), carbamide (CH_4_N_2_O), polyethylene glycol (PEG-400), ethylene glycol (EG), ammonium molybdate ((NH_4_)_6_Mo_7_O_24_·4H_2_O), thiourea (CH_4_N_2_S) and anhydrous ethanol were provided by Sinopharm Chemical Reagent Limited Corporation. All the materials and reagents used in this experiment were of analytical grade and deionized water was used throughout the process.

### 2.2. Synthesis of hollow Fe_3_O_4_

The synthesis of hollow Fe_3_O_4_ was achieved by a facile high-temperature reaction. Firstly, 2.0 g FeCl_3_·6H_2_O, 2 g CH_4_N_2_O, 2 g PEG-400 and 70 ml EG were mixed in a glass beaker, and stirred for a while by a magnetic stirrer until the solid substance was completely dissolved and the solution became transparent orange. Then all the liquid was transferred into a 100 ml Teflon liner equipped with a stainless steel reactor and the temperature was maintained at 200 °C for 16 h. After the product was cooled to normal temperature, it was collected by a magnet and washed with deionized water and anhydrous ethanol three times. Finally it was dried at 60 °C and ground ready for further use.

### 2.3. Synthesis of hollow-structured flower-like Fe_3_O_4_@MoS_2_

The synthesis process of the Fe_3_O_4_@MoS_2_ composite was as follows: a certain amount of Fe_3_O_4_ powder, (NH_4_)_6_Mo_7_O_24_·4H_2_O and thiourea were dissolved in 60 ml deionized water, and then the mixture was transferred into a 100 ml Teflon liner and kept at 180 °C for 12 h. The product was collected by centrifugation and washed with deionized water and anhydrous ethanol three times. At last, it was dried at 60 °C and ground ready for further use. Here, we obtained multiple sets of target products by adjusting the molar ratio of the two materials, and denoted them as T3, T4, T5. Meanwhile, T1 and T2 represented pure Fe_3_O_4_ and MoS_2_, respectively. Table 1 presents the detailed data.

**Table 1:** The different molar ratios between the two materials

Material	T1	T2	T3	T4	T5
Fe_3_O_4_ (mmol)	Pure Fe_3_O_4_	—	0.34	0.34	0.34
Mo precursor (mmol)	—	Pure MoS_2_	3.40	6.12	8.50
Fe_3_O_4_@MoS_2_	—	—	1 : 10	1 : 18	1 : 25

### 2.4. Characterization

Here, X-ray diffraction (XRD; Rigaku Ultimate IV, scanning angle from 10° to 80°) and X-ray photoelectron spectroscopy (XPS; Thermo Scientific K-Alpha) were utilized to characterize the compositions of materials. The surface morphology was investigated by scanning electron microscopy (SEM; Hitachi High-Technologies-4800) and transmission electron microscopy (TEM; JEOL JEM 2100) imaging. Simultaneously, the magnetic property was measured by hysteresis loop (VSM, Lake Shore 7404).

**Fig. 1:**
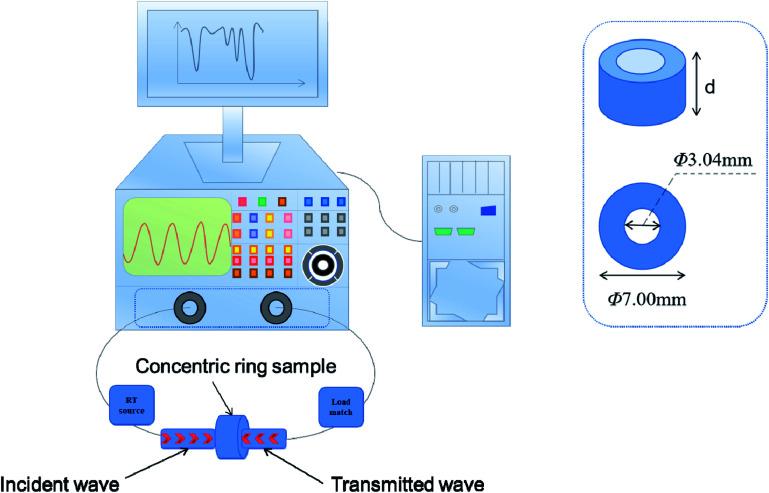
Sketch of the vector network analyzer (VNA).

The electromagnetic parameters were obtained with a vector network analyzer (VNA; N5224A), which measured bands in the 2–18 GHz range. A diagram of the VNA is shown in Fig. 1. Firstly, powders of samples were mixed with paraffin at 1 : 1 mass ratio. Then each was compressed into a concentric ring with a specified size (*Φ*_out_ = 7 mm, *Φ*_in_ = 3.04 mm) by a standard mold. Finally, the data of the samples were calculated by the analysis software and simulated the electromagnetic parameters of the samples under different thicknesses.

## 3. Results and discussion

### 3.1. Mechanism of structure formation

**Fig. 2:**
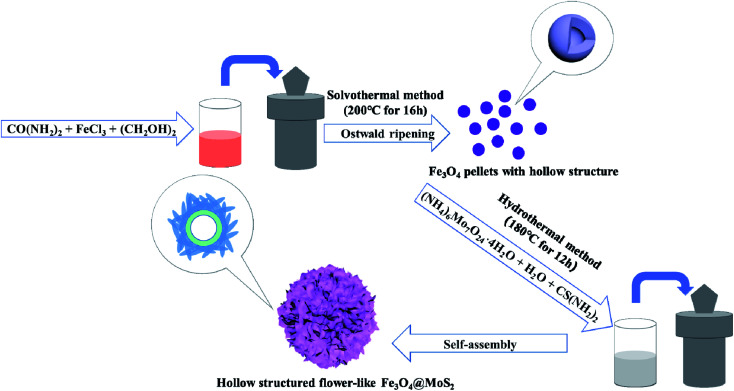
The formation process of a hollow-structured Fe_3_O_4_/MoS_2_ flower-like composite.

The formation process of the hollow Fe_3_O_4_/MoS_2_ flower-like structure composite is shown in Fig. 2. Firstly, Fe_3_O_4_ pellets with hollow structure were prepared by a solvothermal method. The synthesis route is revealed as follows:

1 6CO(NH_2_)_2_ + 6H_2_O + 2FeCl_3_ → C_3_N_6_H_6_ + 3CO_2_↑ + 6NH_4_Cl + 2Fe(OH)_3_↓

2 2HO–CH_2_–CH_2_–OH → 2CH_3_CHO + 2H_2_O

3 2Fe(OH)_3_ + CH_3_CHO → 2Fe(OH)_2_↓ + CH_3_COOH + H_2_O

4 2Fe(OH)_3_ + Fe(OH)_2_ → Fe_3_O_4_↓ + 4H_2_O

The hollow structure can be explained by the Ostwald ripening mechanism. During the reaction, the growth of the nanospheres is due to the combination of the grains. It is well known that the chemical potential of a particle decreases with an increase of particle size, which results in the energy of the internal grains being greater than nanospheres.^23,24^ Finally, under the action of high temperature, grains gradually dissolve and diffuse to the surface of the sphere to form again. At this time, the energy of nanospheres reaches the lowest value, and the hollow structure is formed. Then the MoS_2_ nanosheets were formed on the surface of the spheres. The reaction equations are as follows:^25^

5 CS(NH_2_)_2_ + 2H_2_O → 2NH_3_↑ + CO_2_↑ + H_2_S↑

6 4(NH_4_)_6_Mo_7_O_24_·4H_2_O + 63CH_4_N_2_S + 42H_2_O → 150NH_3_↑ + 63CO_2_↑ + 7H_2_SO_4_ + 28MoS_2_↓

The process of the hydrothermal method is similar to that of crystallization in nature. Herein, the cations on the surface of Fe_3_O_4_ and the anions in molybdate solution attract each other due to the Coulomb force, and the MoS_2_ crystal nucleus is formed at the growth site. With the diffusion of ions to the surface of crystal nucleus and deposition, the crystal will grow directionally along the specific direction and form a unique morphology.

### 3.2. Structural properties

**Fig. 3:**
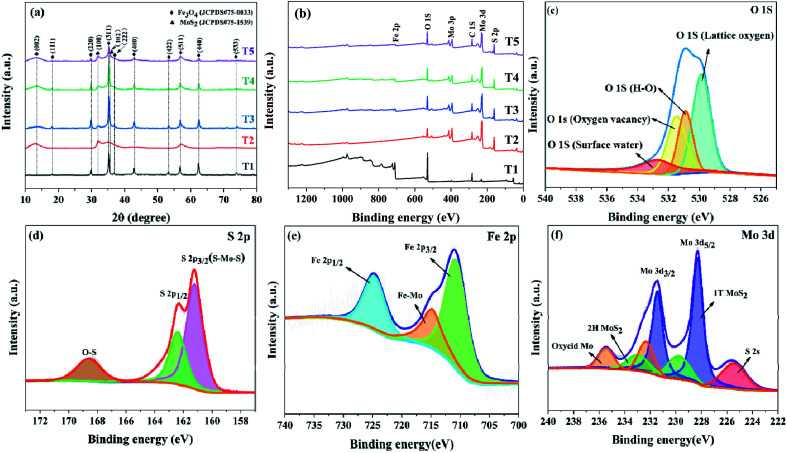
(a) XRD patterns and (b) XPS survey spectra of samples T1, T2, T3, T4, and T5. (c) O 1s, (d) S 2p, (e) Fe 2p and (f) Mo 3d spectra of composite T4.

The characterization of crystal structure is conducive to analyze phase components and purity. Fig. 3a shows the XRD patterns of the samples (T1–T5). By referring to Fe_3_O_4_ (JCPDS no. 75-0033) and MoS_2_ (JCPDS no. 75-1539) standard cards, we can see that the strong diffraction peaks of pure Fe_3_O_4_ (T1) correspond to the face-centered cubic structure planes of (1 1 1), (2 2 0), (3 1 1), (2 2 2), (4 0 0), (4 2 2), (5 1 1), (4 0 0) and (5 3 3). And the principal diffraction peaks of pure MoS_2_ (T2) correspond to the planes of (0 0 2), (1 0 0) and (1 0 2). As for the composite samples (T3–T5), the correlative characteristic peaks of the two component materials are displayed, and the diffraction angle values are at the identical positions. All the patterns have distinct characteristic peaks without impurity peaks, indicating favorable purity and high crystallinity of the products. XPS was used to analyze the surface elements of the samples (Fig. 3b–f) and the full wide-scan spectrum (Fig. 3b) displays the coexistence of Fe, O, Mo and S in the composite. Fig. 3c depicts the O 1s spectrum of composite T4 and it shows four peaks at 529.83 eV, 530.9 eV, 531.6 eV, and 533.06 eV, which correspond to the lattice oxygen, H–O bond, oxygen vacancies and adsorbed water on the surface respectively.^26^ The S 2p spectrum shows two peaks at 161.23 eV and 162.38 eV, which correspond to S 2p_3/2_ and S 2p_1/2_ orbitals respectively. What is more, the peak at 168.58 eV proves the existence of the S–O bond (Fig. 3d). As is shown in Fig. 3e, the two peaks at 710.88 eV and 724.73 eV represent Fe 2p_3/2_ and Fe 2p_1/2_ orbitals while the small peak at 715.13 eV indicates the existence of the Fe–Mo bond. This heterogeneous structure is due to the existence of a large number of atomic vacancies in MoS_2_. In the hydrothermal process, Fe ions diffuse into MoS_2_ at the interface.^27^Fig. 3f shows the Mo 3d spectrum and the two peaks at 228.33 eV and 231.48 eV represent Mo 3d_5/2_ and Mo 3d_3/2_ orbitals. Obviously, the Mo element comes from 1T MoS_2_ (228.38 eV and 231.53 eV), 2H MoS_2_ (229.98 eV and 232.98 eV), and MoO_3_ (235.58 eV and 232.38 eV).^28^ Therefore, the results of XRD and XPS are favorable evidence for the existence of composite.

**Fig. 4:**
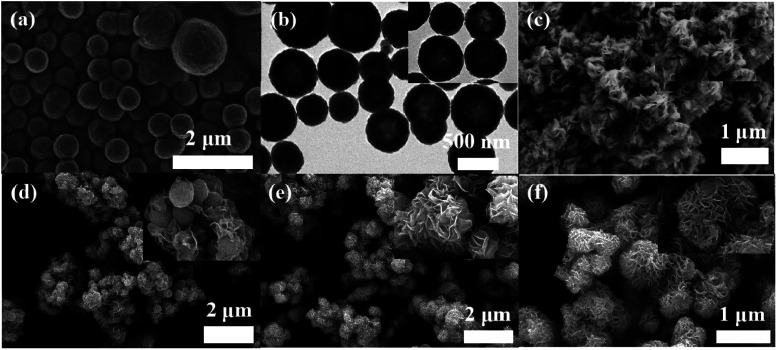
SEM images of sample T1 (a), sample T2 (c), sample T3 (d), sample T4 (e), sample T5 (f) and TEM image of sample T1 (b).

The morphology of a material has a great influence on the microwave-absorbing property. Therefore, SEM and TEM observations are indispensable. Fig. 4a–f show the microscopic morphology of each sample. As for pure Fe_3_O_4_ (T1), the diameter of each nanosphere is about 550 nm, and the size is uniform and the dispersion is good because the solvothermal method provides a stable and mild environment for growth (Fig. 4a). It can be seen from the TEM image that pure Fe_3_O_4_ consists of hollow spheres with outer diameter of 550 nm and inner diameter of 300 nm (Fig. 4b). This unique structure follows the Ostwald ripening mechanism, which not only reduces the system density effectively, but also contributes to the internal multiple reflection loss of EMW.^9^ The pure MoS_2_ (T2) looks like some flowers made of nano-flakes stacked together and it is not very dispersive and has an obvious agglomeration phenomenon (Fig. 4c). It can be predicted that the sheet structure extends in all directions greatly increasing the specific surface area of the material. Then the petal structure grows on the microspheres. As is shown in Fig. 4d, when the molar ratio of Fe_3_O_4_ to Mo precursor is 1 : 10, the nanosheets only grow locally on the microspheres and most of the microspheres are exposed, which indicates the amount of Mo precursor does not reach the ideal proportion. After further increasing the Mo precursor ratio, it can be seen that the surface of the microspheres is completely covered with nanoflakes (Fig. 4e). The composite looks like a flower in full bloom and each microsphere is about 850 nm in size. MoS_2_ nanosheets construct a conductive network around the composite. The dipole polarization generated by the defects and the rapid movement of the polarized electrons provide efficient conduction loss. Meanwhile, the heterogeneous structure of Fe_3_O_4_–MoS_2_ produces interfacial polarization and the interior of the composite provides magnetic loss. Compared to T1 and T2, we can predict that the synergies between the component materials will lead to better microwave absorption.^29^Fig. 4f shows the microstructure of T5; with the increase of Mo precursor, it is difficult for the microspheres to provide enough growth sites on the surface, and the remaining nanosheets can only grow between the petals. In brief, combined with the above structural properties, we successfully prepared hollow-structured flower-like Fe_3_O_4_@MoS_2_.

### 3.3. Microwave properties

**Fig. 5:**
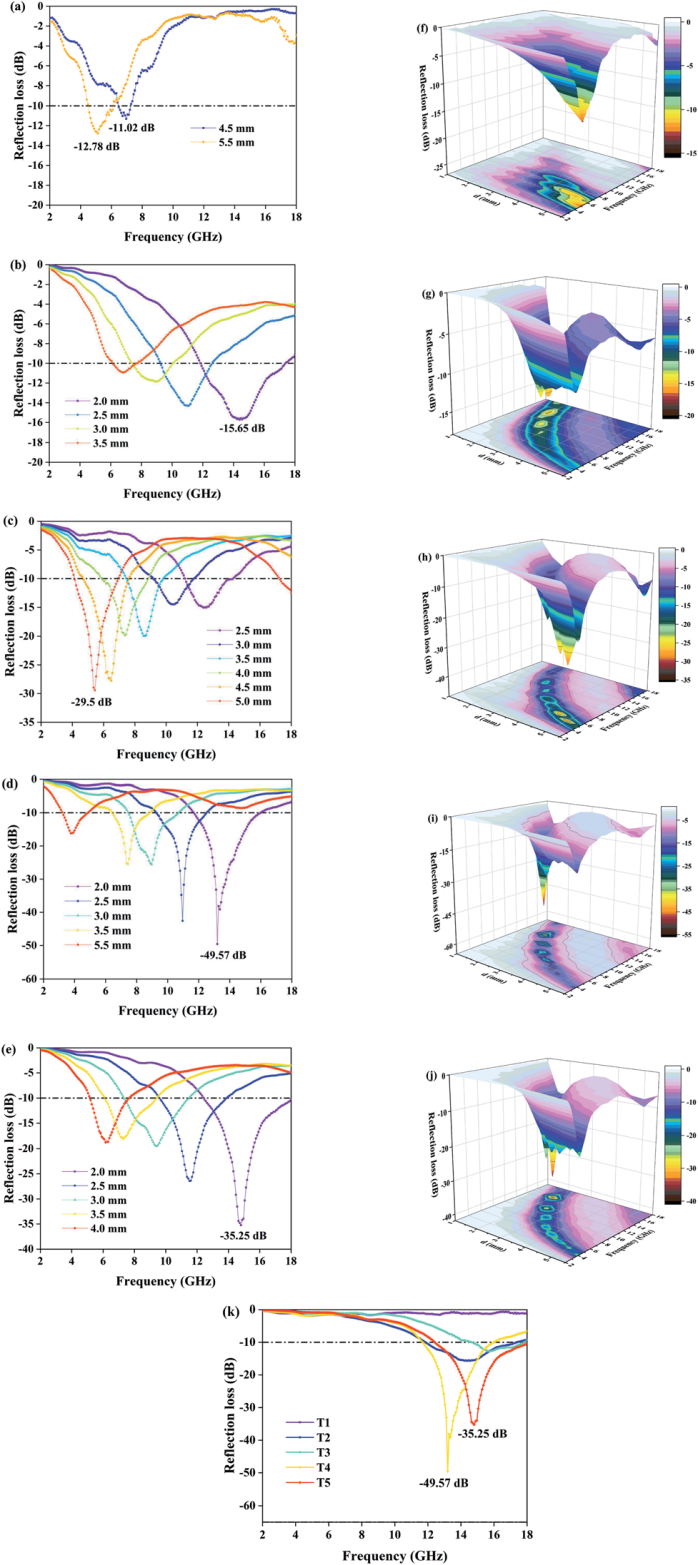
The 2D graphs of measured reflection loss in the frequency range of 2–18 GHz: (a) T1, (b) T2, (c) T3, (d) T4, (e) T5; and the 3D graphs (f–j) revealing the relationship between sample thickness and reflection loss. (k) The 2D reflection loss curve of each sample when the thickness is 2.0 mm.

It is well known that impedance matching and attenuation characteristics determine the absorbing properties of materials. Ideal impedance matching requires the minimum reflectivity of the EMW on a material's surface. The reflectivity is determined by the reflection coefficient (*R*),^30^ the formula being expressed as follows:^31,32^7
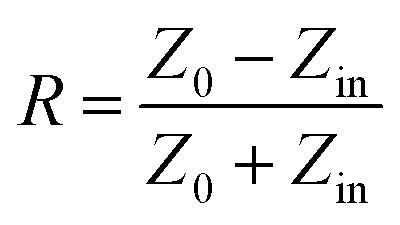
8
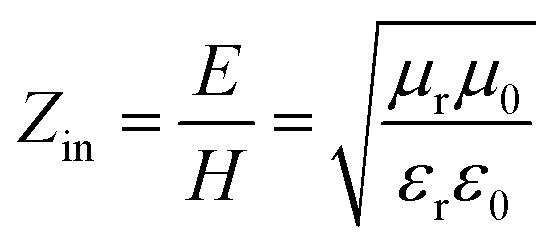
9
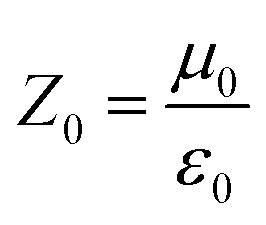
where *Z*_0_ and *Z*_in_ represent the air impedance and the interfacial impedance of the material and *E* and *H* are the electric and magnetic field strengths. They are all related to complex permittivity (*ε*) and complex permeability (*μ*). As mentioned above, when *ε* is equal to *μ*, *Z*_in_ and *Z*_0_ are matched and *R* reaches zero. According to *Z*_in_ and *Z*_0_, we can obtain the following formulas for calculating the reflection loss:^33^10
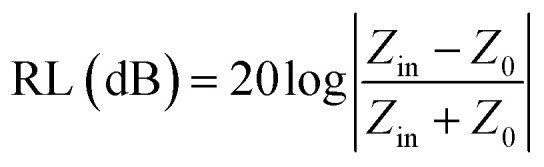
11
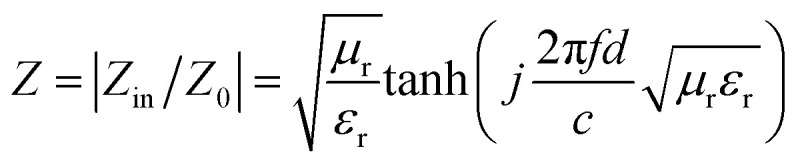
Here, *j* is the imaginary part of a complex number, *f* is the frequency of incident wave, *d* is the thickness of absorbing layer and *c* is the speed of light in a vacuum. The effective EMW absorption band refers to the frequency range when the reflection loss is lower than −10 dB. In this case, we consider that 90% of EMW will be absorbed. The reflection loss diagrams of samples T1–T5 are shown in Fig. 5.

We have selected the obvious absorption curves for each sample and marked the corresponding thicknesses. As for T1 (Fig. 5a and f), the magnetic loss generated by natural resonance means the minimum reflection loss value reaches −12.78 dB at 5.12 GHz with a thickness of 5.5 mm and the effective absorbing bandwidth is 1.52 GHz. Obviously, pure Fe_3_O_4_ has no palpable absorption in other bands except the C band, and the weak absorption capacity, narrow bandwidth and large thickness are not satisfactory. The EMW absorption of pure MoS_2_ (T2) is shown in Fig. 5b and g. Unlike T1, it has distinct absorption peaks in the X and Ku bands. When the thickness is 2.0 mm, the minimum reflection coefficient is −15.65 dB with effective absorption of 5.2 GHz (11.84–17.04 GHz). It exhibits wide bandwidth and small thickness, and can achieve effective absorption within 5.28–18 GHz by designing the thicknesses. Fig. 5c–e show the absorption of composites T3, T4, and T5. For T3, the absorption performance is significantly improved and the minimum reflection loss value reaches −29.50 dB at 5.44 GHz, which indicates that MoS_2_ plays a positive role. Effective absorption can be achieved in the range of 4.16–14.24 GHz when the thickness is changed in the range of 2.5–5.5 mm. However, the improvements of effective absorbing bandwidth and thickness are limited. With an increase of Mo precursor, the maximum absorption peak moves towards high frequency. As displayed in Fig. 5d, the reflection loss of T4 is −49.6 dB at 13.2 GHz and the effective absorbing bandwidth is 4.24 GHz (11.68–15.92 GHz) when the thickness is only 2.0 mm. With an increase of thickness, the sample still maintains outstanding reflection loss. At a thickness of 2.5 mm, 3.0 mm and 3.5 mm, the effective absorption bandwidth is 3.2 GHz (9.28–12.48 GHz), 3.12 GHz (7.52–10.64 GHz) and 2.32 GHz (6.48–8.8 GHz), respectively. The EMW absorption capacity could not continue to increase with the addition of Mo precursor. The minimum reflection loss value of T5 is −35.25 dB at 14.8 GHz. This is because the growth of excessive MoS_2_ among adjacent petals alters the flower-like structure and affects multiple reflection loss. Moreover, the increase of dielectric constant destroys the electromagnetic balance. Surprisingly, the effective absorbing bandwidth is 5.52 GHz (12.48–18 GHz). In the process of adjusting the sample thickness from 2.0 mm to 4.0 mm, it was found that the product could achieve effective absorption in the frequency band 5.2–18 GHz, which contains the X band, the Ku band and most of the C band. The reflection loss curve of each sample with a thickness of 2.0 mm is depicted in Fig. 5k, which shows that the microwave absorption intensity and effective absorption bandwidth of composites T4 and T5 were much better than those of single-material T1 and T2 under the condition of relatively small thickness. Although sample T4 has the highest microwave absorption intensity, the effective absorption bandwidth is slightly smaller than that of sample T5, which is attributed to the addition of excessive Mo precursor. In addition, the absorption curves could shift to the low-frequency direction during the process of increasing the sample thickness. This variation can be explained by the quarter-wavelength matching theoretical equation:^34^12
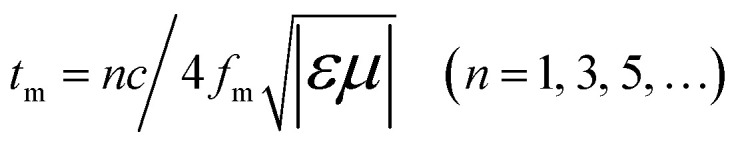
where *t*_m_ is the sample thickness and *f*_m_ is the corresponding frequency. When the value of *n* is 1 and *c* is the constant speed of light, *t*_m_ is inversely proportional to *f*_m_. Therefore, we can acquire strong absorption in corresponding frequency band by designing an appropriate thickness. However, it is difficult to achieve both strong absorption and small thickness in the low-frequency region.

**Fig. 6:**
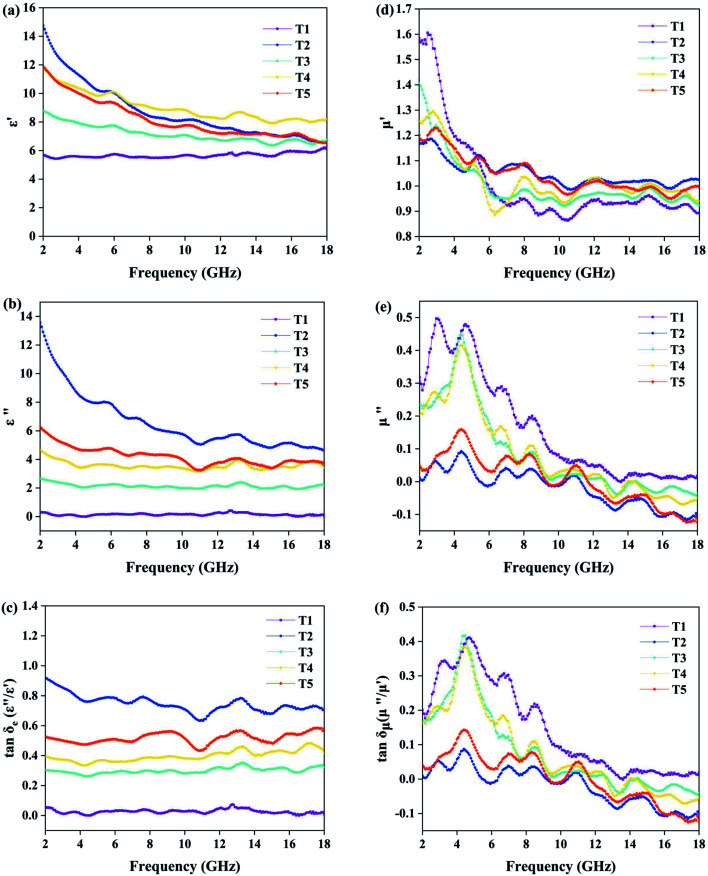
Plots of the real part of the permittivity (a) and permeability (d), the imaginary part of the permittivity (b) and permeability (e) and the tangent of dielectric loss (c) and magnetic loss (f) for samples T1–T5.

The microwave attenuation mechanism is closely related to electromagnetic parameters, which can be expressed by the following formula:^35^13
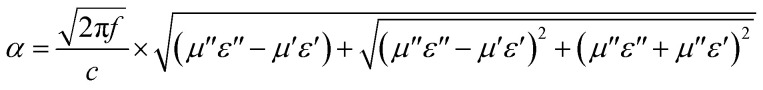
where *α* is the attenuation constant, *f* is the frequency, *c* is the speed of light in vacuum, *μ*′ and *ε*′ represent the real part of the dielectric constant and permeability, and *μ*′′ and *ε*′′ represent the imaginary part of dielectric constant and permeability. It is widely known that the real part of electromagnetic parameters indicates the EMW storage capacity, and the imaginary part of electromagnetic parameters represents the EMW loss capacity. In order to explore the mechanism of loss, we calculate the dielectric constant, permeability and the tangent of loss. The relevant data are shown in Fig. 6.

As a high dielectric loss material, MoS_2_ has the highest values of *ε*′ and *ε*′′ among the five sets of samples, and the curve has an obvious downward trend with an increase of frequency. However, Fe_3_O_4_ is completely opposite to MoS_2_: it has the lowest values of *ε*′ and *ε*′′ and the variation of curves is not significant at 2–18 GHz. As shown in Fig. 6a–c, when increasing the ratio of Mo precursor from T3 to T4, the dielectric coefficient and dielectric loss of the composites are increased. This is due to the leading role of MoS_2_ nanosheets in the construction of an electron transport network. We know that the rapid transfer of electrons contributes to conduction loss and the nanosheets provide channels for electron conduction. As shown in Fig. 6b and c, *ε*′′ and tan *δ*_*ε*_ have similar curve distributions, which indicates that *ε*′′ can reflect the dielectric loss indirectly. Fig. 6d–f show the complex permeability and magnetic loss. We can see that pure Fe_3_O_4_ (T1) and pure MoS_2_ (T2) have the highest and lowest values of *μ*′ and *μ*′′, respectively. With an increase of Mo precursor ratio, the magnetic loss of the samples decreases gradually. Comparing with the variation of complex permittivity, we find that Fe_3_O_4_ has the characteristics of high magnetic loss and low dielectric loss, while MoS_2_ has those of low magnetic loss and high dielectric loss. Therefore, by controlling the ratio, a composite with double loss mechanism can be obtained and the electromagnetic parameters can be balanced. Besides, there are some resonance peaks located at 2–8 GHz for all samples (Fig. 6e and f). Magnetic loss is divided into hysteresis loss, eddy-current loss and residual loss. Since natural resonance exists at relatively low frequency, we consider that these characteristic peaks are caused by natural resonance.

**Fig. 7:**
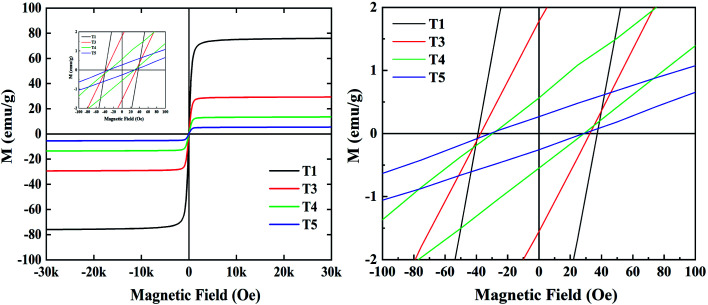
*M*–*H* curves of Fe_3_O_4_ and the Fe_3_O_4_@MoS_2_ composites.

In order to better understand the magnetic property of samples T1, T3, T4, and T5, hysteresis loop tests were carried out and the results are shown in Fig. 7. The data of saturation magnetization and coercivity are exhibited in Table 2.

**Table 2:** The saturation magnetization and coercivity of Fe_3_O_4_ with different ratios of MoS2

Sample	Saturation magnetization (emu g^−1^)	Coercivity (Oe)
T1	75.95	37.5
T3	29.36	32.5
T4	13.52	28.6
T5	5.46	30.9

We know that high saturation magnetization is usually accompanied by high initial permeability and the high initial permeability leads to high magnetic loss. As is shown in Table 2, pure Fe_3_O_4_ (T1) exhibits the highest saturation magnetization of 75.95 emu g^−1^ with the highest coercivity of 37.5 Oe, while saturation magnetization of 29.36, 13.52, and 5.46 emu g^−1^ and coercivity of 32.5, 28.6, and 30.9 Oe are found for T3, T4 and T5. It follows that the magnetic loss of samples can be arranged in the following order: T1 > T3 > T4 > T5; the result is consistent with the curves in Fig. 6f. In addition, the dielectric loss of samples shown in Fig. 6c can be ranked in the following order: T2 > T5 > T4 > T3 > T1; and according to Fig. 5a–j, the order of absorption intensity is T4 > T5 > T3 > T2 > T1. The three different sets of results suggest that remarkable absorbing capacity is not determined by a single loss mechanism, but by the synergistic effect of multiple loss mechanisms.

**Fig. 8:**
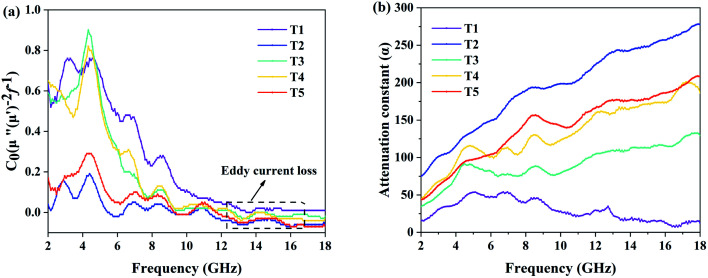
The magnetic eddy current (a) and attenuation constant (b) for samples T1–T5.

The magnetic loss of ferrite is caused by resonance and eddy current loss. The *C*_0_ (*C*_0_ = *μ*′′(*μ*′)^−2^*f*^−1^) values of the samples are calculated from the magnetic permeability and the curves are drawn. In Fig. 8a, each sample has some obvious vibration peaks in the frequency range of 2–10 GHz. Considering that natural resonance generally occurs in the lower frequency band, it can be considered that it is caused by natural resonance. In addition, when the *C*_0_ value of the sample remains constant, the magnetic loss at this time is considered to be eddy current loss. Fig. 8a shows that in the frequency range of 10–18 GHz, *C*_0_ values of all curves tend to flatten out or even stop changing, so there is eddy current loss in the sample.

In order to further illustrate the microwave attenuation capacity of the samples, the attenuation constant (*α*) of each sample was calculated and the results are shown in Fig. 8b. According to the formula of the attenuation constant, an increase of *μ*′′, *ε*′′ and frequency is beneficial to increase its value. In samples T1–T5, the attenuation constant of sample T1 is the smallest, which is consistent with the reflection loss result. And the curve increases first and then decreases, because T1 has the highest *μ*′′ at low frequencies. Surprisingly, the highest attenuation constant was found for sample T2, which reached a maximum of 277 at a frequency of 18 GHz. The attenuation constant curves of samples T2–T5 showed an upward trend on the whole, but fluctuated slightly due to the influence of *μ*′′. In general, a good attenuation constant value should be accompanied by an excellent microwave loss capacity. However, compared with samples T4 and T5, the reflection loss value of sample T2 is obviously lower. This indicates that the attenuation constant is not the only factor affecting the loss capacity of microwaves.

**Fig. 9:**
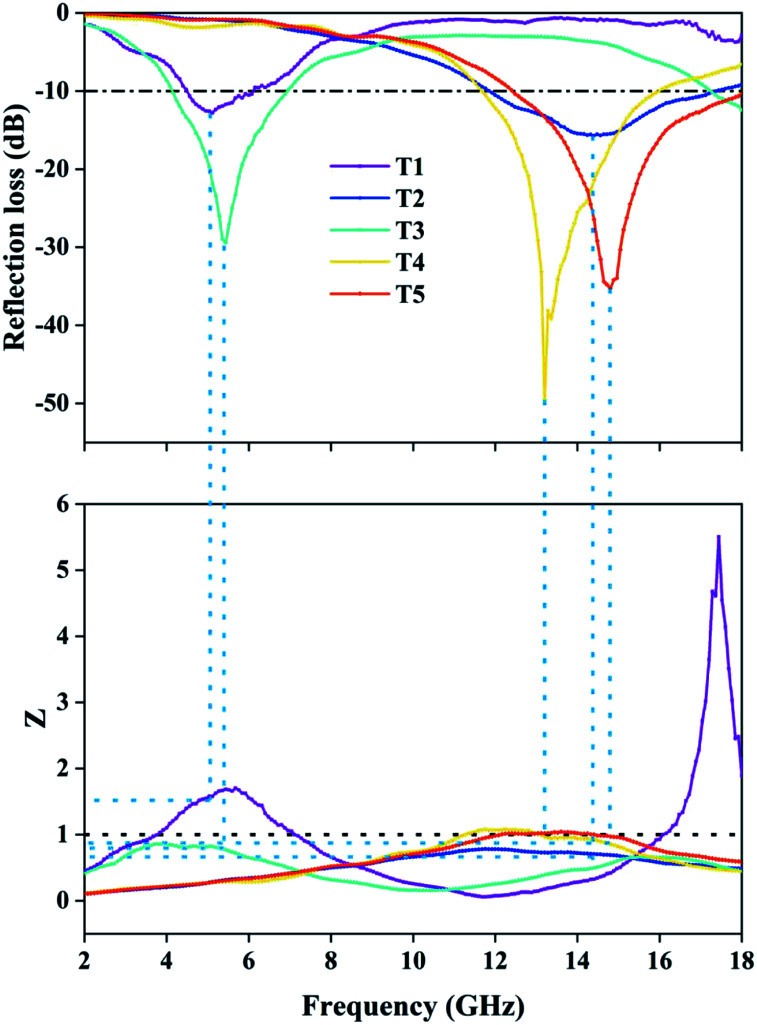
The impedance modulus corresponding to the minimum reflection loss curve of each sample.

As mentioned earlier, attenuation and impedance matching are important factors in determining microwave loss ability. Fig. 9 shows the impedance modulus corresponding to the maximum absorption curves of each sample (T1–T5). We know that when *Z* = 1, a material reaches impedance matching. The corresponding impedance moduli of T4 and T5 at frequencies of 13.2 GHz and 14.8 GHz are 0.975 and 0.961, which are almost a perfect match. As for T1, T2 and T3, their corresponding impedance moduli at frequencies of 5.12 GHz, 14.4 GHz and 5.44 GHz are 1.576, 0.7058 and 0.7686, respectively. Obviously, the combination of MoS_2_ and Fe_3_O_4_ effectively adjusts the electromagnetic parameters, which leads to the Fe_3_O_4_@MoS_2_ composite having better impedance matching than single materials. Although T2 has the largest attenuation constant, poor impedance matching has a negative effect on its microwave absorption ability.

**Fig. 10:**
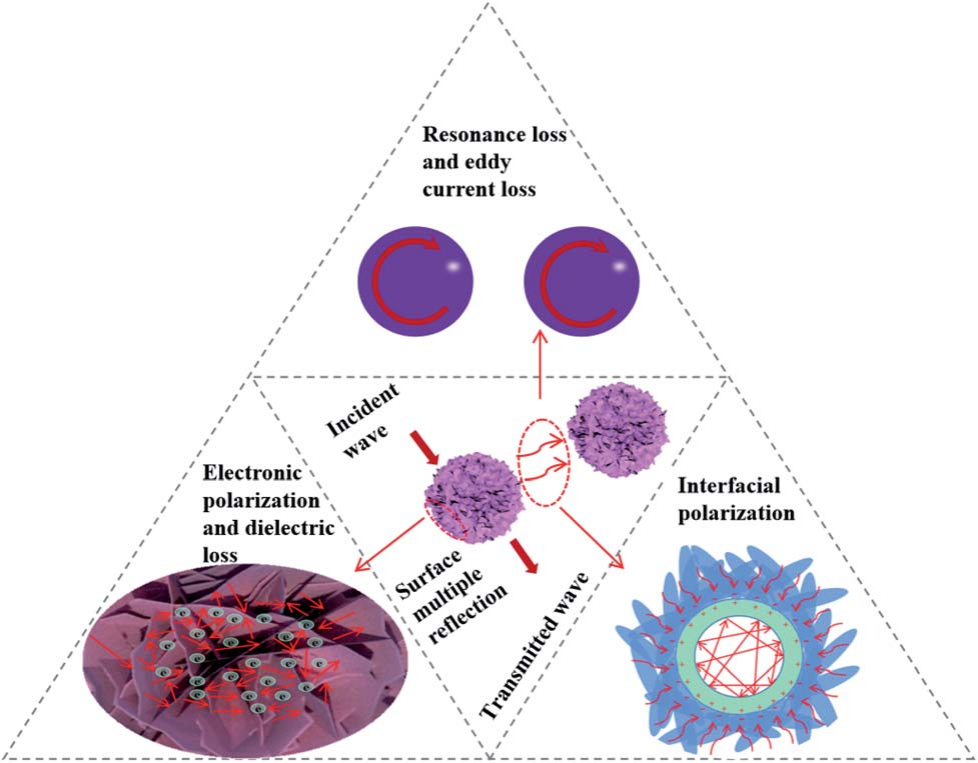
Loss model of EMW in hollow core@shell structured flower-like Fe_3_O_4_@MoS_2_.

In summary, the three composite samples exhibit better absorption ability, effective bandwidth and thickness than each single component. The reason is attributed to the unique construction, the suitable impedance matching and double loss mechanism. The loss model of EMW is shown in Fig. 10. First, the flower-like surface which is stacked by MoS_2_ sheets greatly increases the specific surface area of the material and enables it to receive EMW from all directions. The multiple reflection of incident waves is one of the most significant factors to remove EMW, which occurs not only between the adjacent nanosheets, but also between each flower-like nanosphere.^24^ Second, under a high-frequency electromagnetic field, there are a large number of atomic vacancies on MoS_2_ nanosheets to generate dipole polarization, and the polarized electrons that gain energy move toward the inner core. Third, the heterostructure of Fe_3_O_4_@MoS_2_ is conducive to the accumulation of free charge, which makes each Fe_3_O_4_ sphere negatively charged outside and positively charged inside, resulting in the phenomenon of interfacial polarization and a great loss of electromagnetic energy.^36^ Finally, the residual EMW enter the sphere and disappear as heat under the action of resonance and eddy current loss. Meanwhile, the internal hollow structure causes multiple reflections of EMW, which accelerates the loss of electromagnetic energy.

The excellent loss property and good impedance matching mean that the Fe_3_O_4_@MoS_2_ composite has remarkable absorption strength (−49.6 dB) and satisfactory effective absorption bandwidth (4.24 GHz) at an extremely small thickness. In recent years, a lot of research has been done on Fe_3_O_4_-based materials, and Table 3 lists results for some other absorbers. Through comparison, it is found that the Fe_3_O_4_@MoS_2_ composite is expected to be an outstanding microwave-absorbing material.

**Table 3:** Comparison of the microwave absorption performance of some Fe_3_O_4_-based absorbers

Absorber	RL_min_ (dB)	Thickness (mm)	Effective absorption bandwidth (GHz)	Ref.
Nano-Fe_3_O_4_@C	−46.4	3.5	5.04	37
CNTs-loaded Fe_3_O_4_	−35.9	1.5	4.32	38
Fe_3_O_4_@polyaniline@MnO_2_	−14.7	3.5	4.75	39
ZnO/Fe_3_O_4_	−36.2	2.7	4.02	40
MoS_2_@Ppy@Fe_3_O_4_	−32	2.0	4.3	41
rGO–Fe_3_O_4_	−34.4	1.6	3.8	42
	−37.5	6.5	1.9	
T3	−29.5	5.5	2.72	This work
T4	−49.6	2.0	4.24	
T5	−35.25	2.0	5.52	

## 4. Conclusion

In short, a hollow-structured flower-like Fe_3_O_4_@MoS_2_ composite was successfully prepared by simple solvothermal and hydrothermal methods. Compared with Fe_3_O_4_ and MoS_2_, excellent impedance matching and synergies between materials play a positive role, with the composite exhibiting strong absorption, broad bandwidth and small thickness. In particular, sample T4 has the highest absorption intensity of microwaves, its reflection loss is −49.57 dB at 13.2 GHz and the effective absorbing bandwidth is 4.24 GHz (11.68–15.92 GHz) when the thickness is only 2 mm. Effective absorption in the entire X-band (8–12 GHz) and part of the C-band (4–8 GHz) and Ku-band (12–18 GHz) can be achieved by designing the sample thickness. Sample T5 has the largest effective absorption bandwidth. When the thickness is 2 mm, the effective absorption bandwidth is 5.52 GHz (12.48–18 GHz), and the minimum reflection loss value is −35.25 dB. By adjusting the sample thickness, effective absorption in the 5.2–18 GHz frequency band can be realized. In addition, the hollow structure not only effectively reduces the density of material, but also has a positive effect on microwave absorption. It can be predicted that the hollow-structured flower-like composite has a potential application prospect in the field of microwave absorption.

## Funding

The research was supported by National Natural Science Foundation of China (51703123), the Talent Program of Shanghai University of Engineering Science, and the Shanghai Engineering Research Center for Clean Production of Textile Chemistry (19DZ2253200).

## Author contributions

Guanghong Xiang: writing and idea, Yong Shen: reviewing, Lihui Xu: supervision, Zhewei Ni: data curation, Mingyang Chen: software.

## Conflicts of interest

The authors declare no conflict of interests.

## Supplementary Material
